# Syndromic Disorders Caused by Disturbed Human Imprinting

**DOI:** 10.4274/jcrpe.galenos.2019.2018.0249

**Published:** 2020-03-19

**Authors:** Diana Carli, Evelise Riberi, Giovanni Battista Ferrero, Alessandro Mussa

**Affiliations:** 1 University of Torino, Department of Pediatric and Public Health Sciences, Torino, Italy

**Keywords:** Imprinting disorders, epimutation, genotype, phenotype

## Abstract

Imprinting disorders are a group of congenital diseases caused by dysregulation of genomic imprinting, affecting prenatal and postnatal growth, neurocognitive development, metabolism and cancer predisposition. Aberrant expression of imprinted genes can be achieved through different mechanisms, classified into epigenetic - if not involving DNA sequence change - or genetic in the case of altered genomic sequence. Despite the underlying mechanism, the phenotype depends on the parental allele affected and opposite phenotypes may result depending on the involvement of the maternal or the paternal chromosome. Imprinting disorders are largely underdiagnosed because of the broad range of clinical signs, the overlap of presentation among different disorders, the presence of mild phenotypes, the mitigation of the phenotype with age and the limited availability of molecular techniques employed for diagnosis. This review briefly illustrates the currently known human imprinting disorders, highlighting endocrinological aspects of pediatric interest.

## Introduction

The imprinting disorders are a group of congenital diseases caused by dysregulation of genomic imprinting that can affect fetal and postnatal growth, neurocognitive development, metabolism and cancer predisposition with relevance to pediatricians, geneticists, endocrinologists and other specialists (1,2,3,4,5,6). Genomic imprinting mediates the expression of specific genes in a parent of origin specific manner. While most genes are expressed biparentally, imprinted genes are expressed only from the paternal or the maternal allele. Imprinted genes are often arranged in clusters and expressed under a coordinated epigenetic regulation (4,7). Human imprinting disorders result from dysregulation of the normal expression of imprinted genes, causing altered dosage or function of such gene transcripts. This can be achieved through different mechanisms, which may involve DNA expression only (epigenetic mechanisms) or may also encompass DNA sequence (genomic mechanisms). While the former are mostly sporadic, the latter result in familial forms with a parent of origin inheritance pattern (5).

**Figure 1:**
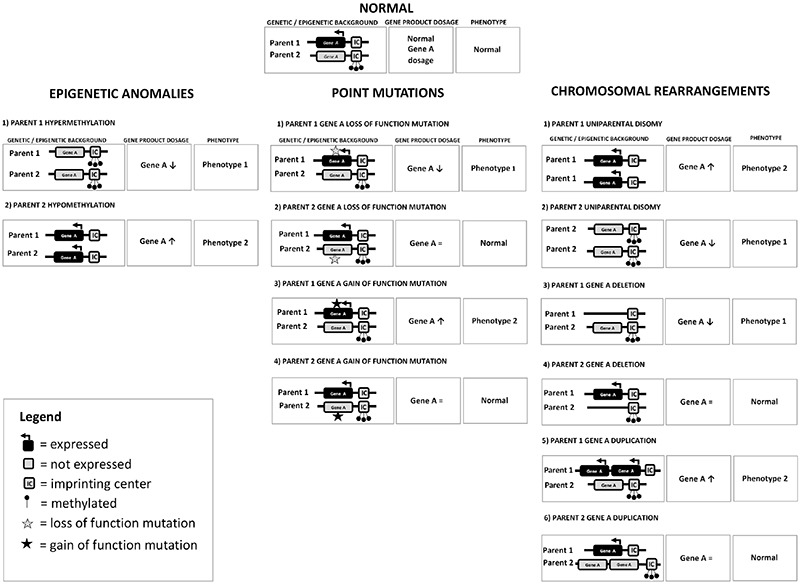
Schematic representation of the molecular mechanisms responsible for altered imprinted gene expression. At the top normal functioning of a paradigmatic chromosomal region subjected to imprinting is reported: on the allele inherited from parent 1, the imprinting center (IC) is unmethylated and gene A is expressed, while on the allele inherited from parent 2, gene A is silenced by IC methylation. This leads to a balanced expression of gene A, corresponding to the normal phenotype. Conversely, imbalance between the expression of the imprinted gene leads to a pathological phenotype: a deficiency of gene A leads to phenotype 1, while an excess of gene A leads to phenotype 2. Phenotype 1 and phenotype 2 may have antithetical characteristics (mirror phenotypes). In the left column, epigenetic anomalies leading to disturbed expression of imprinted genes are shown. In the middle column, point mutations and in the right column, uniparental disomy, deletion and duplication affecting the imprinted gene are reported. If the point mutation or the deletion/duplication hits the expressed gene, it will lead to a phenotype while, on the opposite, if they involve a normally silenced gene, they will not result in a phenotype: in both cases, the genetic anomaly could be transmitted to the offspring

The molecular mechanisms responsible for altered imprinted gene expression (Figure 1) are classified into:

1. Uniparental disomy (UPD), which consists of the inheritance of two copies of a chromosome (or part of a chromosome) from one parent and no copy from the other parent. UPD can be heterodisomy, when both homologue chromosomes from the transmitting parent are present, or isodisomy, when two identical chromosomes from the same parental homologue are present (8).

2. Abnormal methylation (also termed epimutation) including excessive methylation (hypermethylation or gain of methylation - GoM) and reduced methylation [hypomethylation or loss of methylation (LoM)]. Abnormal methylation can be primary (i.e. in the absence of an underlying genomic cause) or secondary (i.e. due to an underlying genomic cause). While the former is sporadic, the latter is associated with a recurrence risk, in an autosomal dominant manner with parent of origin effect.

3. Chromosomal abnormalities (deletions, duplications and balanced rearrangements).

4. Intragenic variants in imprinted genes resulting in loss or gain of function.

For all these four mechanisms, the phenotype depends on the affected parental allele; in some cases, aberrations at the same locus involving either the maternal or the paternal chromosome result in opposite phenotypes (Table 1). Although each imprinting disorder is characterized by specific clinical features, shared phenotypic features are common and clinical overlap occurs. Moreover, mild phenotypes, a broad clinical spectrum, mitigation of the presentation with age and limited availability of the molecular techniques employed for diagnosis probably lead to a relevant underdiagnosis (4,5).

Most patients with an imprinting disorder are affected by a single disease-specific locus with a definite phenotype. However, cases with multilocus methylation imprinting disturbances (MLID) and consequent complex phenotypes are increasingly described and further complicate the clinical evaluation. Of interest, the frequency of some of the imprinting disorders is increased in the offspring of subfertile parents and likely connected with artificial reproductive techniques (9,10).

Recent advances in this field suggest that the range of imprinting disorders could be greater than those currently described. In this article we review those described hitherto, ordered by chromosome.

## Chromosome 6

### Transient Neonatal Diabetes Mellitus Type 1

Transient neonatal diabetes mellitus type 1 (TNDM1, OMIM #601410) has a prevalence of approximately 1 in 500,000 births (11) and it is characterized by intra-uterine growth restriction (IUGR) and infantile hyperglycemia in the absence of ketoacidosis. Macroglossia and umbilical hernia are often present. TNMD1 features are evident in infants during the first weeks of life, usually presenting with dehydration, and generally disappearing by the age of 18 months. Insulin treatment is usually required. However, diabetes may relapse later in life in approximately half of the patients, showing characteristics of type 2 diabetes mellitus. Women may relapse during pregnancy presenting with gestational diabetes mellitus (12).

TNDM1 can be caused by three different molecular mechanisms (12):

1. Paternal UPD of chromosome 6 (41%).

2. Duplication of the paternal allele at 6q24 (29%).

3. Hypomethylation of the maternal differentially methylated region (DMR), *PLAGL1: alt-TSS-DMR *(30%).

This latter mechanism can be due to either an isolated imprinting variant or as part of a generalized hypomethylation at imprinted loci (MLID), due to recessive loss of function *ZFP57* mutations in almost half of the cases (13). TNDM1- MLID patients may have further phenotypic manifestations, such as structural brain abnormalities, developmental delay and congenital heart disease (14).

All three molecular mechanisms accounting for TNDM1 lead to over-expression of the *PLAGL1/ZAC* gene which regulates apoptosis and cell cycle arrest (15). The protein encoded by the *PLAGL1/ZAC* gene is a zinc finger protein and regulates *PACAP1* that has a key role in stimulating insulin secretion by pancreatic beta cells. Moreover, *PLAGL1/ZAC1* gene overexpression may reduce the number of beta cells or impair their function, stopping cell cycling and inducing apoptosis (12).

## Maternal Uniparental Disomy of Chromosome 6

Maternal UPD of chromosome 6, abbreviated to UPD(6)mat, has been hypothesized to be associated with IUGR and other heterogeneous clinical features, especially intellectual disability (16). However, homozygosity of a recessive allele and/or placental trisomy 6 mosaicism is likely to be the pathogenic mechanism in some of these patients. These data suggest that a specific imprinting disorder associated with UPD(6)mat does not exist and that the heterogeneous clinical features in UPD(6)mat patients are either caused by placental trisomy 6, undetected trisomy 6 cell lines or by homozygosity for recessive mutations (5,17). However, given the small number of patients described to date and the presence of an imprinted region on chromosome 6q24 further studies are required to clarify this contentious issue.

## Chromosome 7

Maternal UPDs of chromosome 7 are responsible for a small subset (5-10%) of Silver-Russell syndrome (SRS). Since the majority of SRS cases are due to chromosome 11 abnormalities, this topic is extensively described in the chromosome 11 section.

## Chromosome 8

### Birk-Barel Syndrome

Birk-Barel syndrome (OMIM #612292) is characterized by severe neonatal hypotonia, transient neonatal hypoglycemia, joint contractures, wide alveolar ridges, cleft palate, microretrognathia, developmental delay and variable intellectual disability. Distinctive facial features include dolichocephaly, bitemporal narrowing, short philtrum, tented upper lip and medially ﬂared eyebrows (18,19).

This disorder is caused by a speciﬁc missense mutation (c.770G>A, p.Gly236Arg) in the maternal copy of the *KCNK9/TASK3* gene, located in chromosomal region 8q24.

The 8q24 chromosomal region includes two imprinted genes: *PEG13*, expressed by the paternal allele and *KCNK9*, expressed by the maternal allele. The reciprocal expression of these genes is regulated by a maternal methylated region located within the *PEG13* transcript, named *PEG13:TSS-DMR* (20).

The *KCNK9/TASK3 *gene encodes a member of the two pore-domain potassium channel subfamily (18,19). TASK3 channels are widely expressed, especially in the brain, where they play a role in the migration of cortical pyramidal neurons regulating both neuronal activity and neuronal development. Of note, nonsteroidal anti-inﬂammatory fenamic acid drugs, especially ﬂufenamic acid, are able to stimulate the two pore-domain potassium channels, partially rescuing the reduced outward current through mutated *KCNK9* channels, suggesting that fenamic acid compounds might be useful in treating this condition (18).

## Chromosome 11

### Beckwith-Wiedemann Syndrome

Beckwith-Wiedemann syndrome (BWS) (OMIM #130650) is the most common congenital overgrowth condition (1:10,500 live births) (21) and represents the paradigm of genetic imprinting disorders and cancer predisposition syndromes. Clinical features include neonatal macrosomia, postnatal overgrowth, macroglossia, abdominal wall defects ranging from severe (omphalocele, gastroschisis) to moderate (umbilical hernia) and mild (*diastasis recti*), ear pits and creases, glabellar *naevus flammeus*, lateralized overgrowth (previously termed hemihyperplasia) (22), organomegaly, nephroureteral malformations (23), hyperinsulinism or transient hypoglycaemia (1), placental mesenchymal dysplasia and predisposition to the development of embryonal tumors in infancy (24). These features combine variably accounting for the different degree of severity of presentation and depicting a broad phenotypic spectrum (25,26,27,28,29), including cases with isolated lateralized overgrowth (22). The diagnosis is clinical, based on criteria and a scoring system which has been recently revised (1).

BWS is caused by several epigenetic and genetic defects. In approximately 85% of patients disturbed expression of imprinted genes located into two separate domains on chromosome 11p15.5 is found. In this chromosomal region, two differentially methylated imprinting centers (IC) (*H19/IGF2:IG-DMR* and *KCNQ1OT1:TSS-DMR*, commonly referred to as IC1 and IC2, respectively) control the expression of genes involved in cell cycle progression and somatic growth control. Five mechanisms leading to the disruption of the expression of such genes are currently known:

1. Approximately 50% of cases are caused by LoM at IC2 (IC2-LoM) leading to reduced expression of *CDKN1C*, normally expressed by the maternal chromosome only. IC2-LoM is usually a sporadic primary epigenetic defect, however rare familial cases carrying genetic mutations causing secondary hypomethylation have been described (30). An increasingly growing fraction of patients with IC2-LoM also display methylation abnormalities at other imprinted loci leading to additional phenotypes (MLID) (31,32). Disruption of trans-acting mechanisms regulating the normal imprinting at the 11p15.5 ICs as well as other differentially methylated regions can be responsible for such cases; rare inheritable mutations in the *NLRP *family genes have been described (33,34,35). NLRP proteins are members of the NLR family of proteins and are important components of inflammasomes with a major role in innate immunity (36). Interestingly, a subset of *NLRP* genes is expressed in oocytes and early embryos (37). Females with mutations in* NLRP2* and *NLRP7* gave birth to few or no liveborn children (38). Germline mutations in *NLRP2* are responsible for a familial form of BWS caused by a trans-acting mechanism, consistent with the hypothesis that *NLRP2* has a role in establishing or maintaining genomic imprinting in humans (33). *NLRP5* mutations have also been reported in five mothers of offspring with MLID, linking this gene with a maternal effect on reproductive fitness, epigenetic and developmental reprogramming of zygotes and reproductive outcomes (32,39).

2. Mosaic segmental paternal UPD of chromosome 11, accounting for 20% of the cases, leads to altered expression at both gene clusters (1) with IC2-LoM and IC1-GoM. Genome-wide UPD of chromosome 11 is found in a subset of cases and associated with higher cancer risk (40).

3. IC1-GoM results in biallelic expression of the *IGF2 *gene which is normally expressed by the paternal allele only and reduced expression of the *H19* gene, an oncosuppressor gene normally expressed by the maternal allele. IC1-GoM is found in 5-10% of cases and in a subset of patients is caused by microdeletions encompassing the *OCT4/SOX2* binding site localized inside IC1, leading to a maternally transmitted BWS phenotype (41,42).

4. Maternal *CDKN1C* loss-of-function mutations are responsible for maternally inheritable BWS and account for 5-10% of cases.

5. Finally, approximately 1% of BWS cases are caused by chromosomal rearrangements (duplications, translocations, inversions, deletions) involving the 11p15.5 chromosomal region and causing secondary IC1-GoM or IC2-LoM (24).

About 15% of clinically diagnosed BWS cases have no detectable molecular defect when investigated using commonly employed diagnostic molecular techniques. However, low somatic mosaicism of the above mentioned defects is increasingly found by using novel molecular techniques (43) and analysing tissues other than blood (e.g. buccal smear) (44). It cannot be excluded that in a fraction of patients the molecular defect has not yet been discovered.

Besides providing diagnostic confirmation and the possibility of genetic counselling, molecular anomalies detected in BWS have implications for the clinical management of patients and prognostic value. Indeed, specific correlation between epigenotype and phenotypic features are present, especially concerning cancer risk (26,27,28,45). BWS molecular subtypes are characterized by a gradient in cancer development probability and display different histotypes allowing differentiation of tumor surveillance protocols according to the epigenotype. This facilitates the early detection of relevant associated tumors, with special reference to Wilms’ tumor and hepatoblastoma (26,45,46,47,48,49,50,51,52).

## Silver-Russel Syndrome

SRS (OMIM #180860) is the phenotypic and genetic opposite disorder of BWS, has an estimate incidence of 1:30,000 to 1:100,000 (2) and represents the paradigm of genetic restricted growth imprinting disorders and poor feeding predisposition.

The phenotypic clinical spectrum of SRS includes severe IUGR, postnatal growth failure with no catch-up, body hemihypoplasia with body asymmetry, relative macrocephaly with triangular face, typical facial appearance (prominent forehead, narrow chin, small jaw and downturned corners of the mouth), low muscle mass, fifth finger clinodactyly, feeding difficulties, recurrent hypoglycemia, premature adrenarche, rapidly progressing and/or central precocious puberty (CPP) and insulin resistance in adulthood (2,53).

The diagnosis of SRS is clinical and molecular testing is used for confirmation and phenotype stratification. Given the broad spectrum of presentation, the diagnosis is based on the Netchine-Harbison scoring system (54), having high sensitivity and predictive value. A molecular cause can be identified in approximately 60% of patients with a clinical diagnosis (2), while the molecular aetiology remains unknown in a substantial proportion of patients:

1. The most common mechanisms is LoM at IC1 on the paternal chromosome 11p15 (IC1-LoM), which is detected in 40-60% of patients. IC1-LoM results in reduced*IGF2*expression and increased *H19* expression (2,55).

2. Besides IC1-LoM, a variety of rearrangements involving the 11p15.5 region resulting in a SRS phenotype have been described (56,57).

3. From 5 to 10% of cases are caused by maternal UPD of chromosome 7 (2).

4. Mirroring BWS molecular alterations in chromosomal region 11p15.5, the SRS phenotype also results from alterations at the centromeric IC2 of 11p15.5. Genomic imbalances involving IC2 resulting in gain of methylation at this center have been rarely described (58).

5. Rare monogenic causes have been described including a mutation increasing CDKN1C stability in a family with maternally transmitted SRS (59), *IGF2* loss-of-function mutation in a family with paternally transmitted SRS (60) and *HMGA2* and *PLAG1* mutations with dominant transmission regardless of maternal or paternal transmission (61,62,63). Coding variants in these genes are overall very rare (2).

Differential diagnosis of SRS includes other genetic syndromes characterized by growth restriction, including single gene disorders such as IMAGE syndrome (discussed immediately below) and Temple syndrome (discussed in the chromosome 14 section) and chromosomal anomalies and copy number variants (2). The differential diagnosis can have extremely important implications for management since SRS treatment may include growth hormone (GH) therapy (53) and response to treatment. For instance, GH treatment is contraindicated in patients with chromosome breakage disorders due to the associated risk of malignancy (2).

## IMAGE Syndrome

IMAGE syndrome (OMIM #614732) results from a gain-of-function mutation in the *CDKN1C* gene, negatively regulating cellular proliferation. Since *CDKN1C *is expressed only from the maternal allele, IMAGE syndrome occurs only when the *CDKN1C* gain-of-function mutation is inherited from the mother (64). This syndrome is characterized by SRS phenotype associated with metaphyseal dysplasia, congenital adrenal hypoplasia with adrenal insufficiency, and almost always includes genital anomalies (65).

## Chromosome 14

### Temple Syndrome

Temple syndrome (OMIM #616222) is characterized by prenatal and postnatal growth failure and early onset of puberty with final short stature, hypotonia, feeding difficulties in early childhood, motor delay, joint laxity, truncal obesity and minor dysmorphic features such as broad forehead and short nose with wide nasal tip and small hands and feet (66). Due to relatively mild and age-dependent characteristics, the prevalence of Temple syndrome in the general population is unknown and the disorder is likely underdiagnosed in clinical practice (66).

Temple syndrome shows several nonspecific clinical features overlapping with Prader-Willi syndrome (PWS) and SRS (67,68,69). The treatment may include GH therapy (70).

The syndrome is caused by alteration of imprinted gene expression at chromosome 14q32.2. This region contains a cluster of imprinted genes including three paternally expressed genes (*DLK1*, *DIO3* and *RTL1*) and multiple maternally expressed non-coding RNAs (*MEG3*, *RTL1as*, *MEG8*, *snoRNAs*, and *microRNAs)* (71). The parental origin-dependent expression patterns are regulated by a germline-derived primary intergenic DMR (*MEG3/DLK1:IG-DMR*) and a postfertilization-derived secondary DMR (*MEG3:TSS-DMR*), both normally methylated only on the paternal allele (72). Mechanisms that result in functional hemizygosity of 14q32 imprinted genes can cause the clinical phenotypes (4), including:

1. Chromosome 14 maternal UPD (78%) (73).

2. Isolated methylation deficiency at *MEG3:TSS-DMR* in the 14q32.2 imprinted region (12%) (74).

3. 14q32 deletions of paternal origin (10%) (71).

Maternal UPD of chromosome 14 represents the major molecular cause of Temple syndrome. However, some evidence indicate that UPD over-representation among the molecular causes of Temple’s syndrome could be due to an ascertainment bias and it is possible that frequencies of the molecular findings in Temple syndrome will be updated in the coming years (75).

## Kagami-Ogata Syndrome

Kagami-Ogata syndrome (OMIM #608149) includes overgrowth (typically with birth weight disproportionately greater than length), polyhydramnios, placentomegaly, poor sucking and hypoventilation in the neonatal period, abdominal wall defects ranging from omphalocele to *diastasis recti*, a distinctive facial appearance (full cheeks, depressed nasal bridge, micrognathia, short webbed neck and protruding philtrum), small bell-shaped thorax with coat-hanger ribs, and variable developmental delay and/or intellectual disability. Some features are rather nonspecific and can be also observed in BWS. Kagami-Ogata syndrome is associated with increased risk of developing hepatoblastoma (9%) and a neonatal mortality rate as high as 20-25% (76).

Kagami-Ogata syndrome can be caused by three different molecular mechanisms (4):

1. Paternal UPD of chromosome 14 (65%).

2. Microdeletion affecting the maternal 14q32.2 imprinted region (20%).

3. Hypermethylation (15%) affecting the *MEG3:TSS-DMR* in the maternal 14q32.2 imprinted region (77).

While UPD(14)pat and hypermethylation are sporadic, microdeletions can lead to a maternally transmitted Kagami-Ogata syndrome. Recently it has been shown that causal deletions do not necessarily include the DMRs; therefore, a normal methylation pattern does not exclude the syndrome (78).

As discussed for Temple’s syndrome, it has been proposed that over-representation of UPD(14)pat among the molecular causes of the Kagami-Ogata syndrome could be secondary to an ascertainment bias and the frequencies of the molecular causes could change as availability of specific molecular tests increases (75).

## Chromosome 15

### Angelman Syndrome

Angelman syndrome (AS) (OMIM #105830) is characterized by developmental delay, intellectual disability with severe speech impairment, microcephaly and seizures. The symptoms usually appear in the first year of life (79). Seizures typically occur between one and three years of age and can be associated with generalized, specific electroencephalographic changes (80). Patients also present with sleep disruption, excessive laughter, happy demeanor, gait ataxia, tremulousness of the limbs and protruding tongue. AS prevalence is approximately one in 12,000-24,000 live births (80).

AS can be caused by four different mechanisms:

1. Maternally derived *de novo* deletion of 15q11-q13 (70-75%).

2. Paternal UPD of chromosome 15 (3-7%).

3. Imprinting defect at *MKRN3:TSS-DMR* in the maternal chromosome 15q11.2-q13 locus (2-3%).

4. Maternally inherited mutations in *UBE3A* gene (10-15%) (5).

The phenotype is usually more severe in patients with large deletions. All genetic mechanisms result in lack of expression of the maternally expressed 15q11-q13 *UBE3A* gene. *UBE3A* is normally expressed exclusively from the maternal allele in human fetal brain and in adult frontal cortex. Duplications of this gene have been linked to autism spectrum disorder, developmental delay and neuropsychiatric phenotypes (81), further supporting the hypothesis that *UBE3A *plays a pivotal role in neurodevelopment. AS patients have a paternal copy of *UBE3A*, but it is silenced by a nuclear localized long non-coding RNA, known as *UBE3A* antisense transcript (*UBE3A-ATS*) (82). Antisense oligonucleotides treatment aimed at reducing the *UBE3A-ATS* in order to unsilence the paternal *UBE3A *gene is under study (82).

## Prader-Willi Syndrome

PWS (OMIM #176270) includes variable characteristics according to the age of the patient. Decreased fetal movement, abnormal fetal position at delivery, and increased incidence of assisted delivery or cesarean section are common. Hypotonia of central origin with poor sucking and feeding difficulties resulting in failure to thrive are prevalent in the neonatal period and in the first year of life. Subsequently, progressive hyperphagia with central obesity occurs. Hyperphagia is linked to a hypothalamic dysfunction resulting in lack of satiety and food-seeking behavior with central obesity being the result of both hyperphagia and a reduced total energy expenditure connected with decreased physical activity and decreased lean body mass. Extreme obesity and related complications represent the major causes of morbidity and mortality in PWS (83). Hypothalamic hypogonadism with cryptorchidism, incomplete genital development, delayed and incomplete puberty and infertility are typical (84). Short stature is very common and is usually treated with GH replacement therapy, with the additional benefit of acquisition of lean mass. Abnormalities of GH function in PWS have been reported and other hypothalamic hormones can also be deficient causing tertiary hypothyroidism, and central adrenal insufficiency (85). PWS patients may exhibit developmental delay of variable severity. Behavior problems are common and manifest with a typical pattern including temper tantrums, controlling and manipulative behavior and compulsivity. Current trials are underway to evaluate oxytocin as a potential therapeutic agent for controlling behavior issues in PWS patients (86,87).

Characteristic facial features may develop over time and include narrow bifrontal diameter and nasal bridge, almond-shaped palpebral fissures, thin vermilion of the upper lip with down-turned corners of the mouth.

Diagnosis and molecular testing is based on clinical criteria (88).

PWS is caused by lack of expression of imprinted genes on chromosome *15q11.2-q13* gene cluster, defined as the “PWS critical region”. Alterations not involving this critical region are not associated with PWS. The PWS critical region encompasses imprinted genes normally expressed only on the paternal allele: *MKRN3*, *MAGEL2*, *NDN*, *PWRN1*, *C15orf2*, *SNURF-SNRPN* and several snoRNA genes. The deficiency of one of these snoRNA (*SNORD116*) is believed to elicit the key features of PWS phenotype (89,90).

Altered expression can be caused by four mechanisms:

1. Deletion of the 15q11-q13 imprinted loci on the paternal allele (up to 70-75% of cases).

2. Maternal UPD of chromosome 15 (up to 20-25%).

3. Imprinting defects due to primary epimutations at *MKRN3:TSS-DMR* (2%) (84,91).

4. Small deletions within the IC critical region which may or may not lead to an imprinting deficiency detectable by methylation analysis (<0.5%) (84,91,92).

Most PWS cases are sporadic. Inheritable PWS is rare and can be due to deletions caused by unbalanced chromosome rearrangement or paternally inherited IC deletion. The diagnosis is confirmed through DNA methylation analysis, with subsequent cytogenetic testing, fluorescence *in situ* hybridization and microsatellite marker analysis, which define the genotype classifications (93).

## Schaaf-Yang Syndrome

Schaaf-Yang syndrome (OMIM #615547) is a PWS-like disease, due to truncating mutations in the *MAGEL2* gene, which is located in the PWS critical region (chromosome 15q11-q13) and is normally maternally imprinted and paternally expressed. Schaaf-Yang syndrome is characterized by neonatal hypotonia, developmental delay and intellectual disability, hypogonadism, autistic behavior and joints contractures. The typical PWS features of hyperphagia and obesity are usually absent. Consequently, the phenotypic overlap with PWS is preeminent in the neonatal period. The phenotypic spectrum ranges from severe fetal akinesia to mild expression including intellectual disability and finger contractures (94).

Paradoxically, while truncating mutations in the *MAGEL2* gene cause Schaaf-Yang syndrome, *MAGEL2* whole gene deletions cause on slight or even absent expression of the clinical phenotype (94). It is likely, as *MAGEL2 *is a one-exon gene, that truncating mutations may result in a shortened protein with a dominant-negative effect. As an alternative explanation to this phenomenon, the deletion of the entire paternal copy of the gene, including its promoter, could lead to leaky expression of the maternal copy of the gene (94).

## Central Precocious Puberty 2

CPP (OMIM #176400) also known as gonadotropin dependent precocious puberty, is characterized by a premature activation of the reproductive axis, before the age of eight years in girls and nine years in boys (95). Prevalence of CPP has been estimated at approximately 1.1:100,000 with an overall male to female ratio of at least 1:10 (96). Subjects affected by CPP present with pubertal signs such as breast development or testicular enlargement and acceleration of growth and bone age, consistent with elevated basal and GnRH-stimulated LH levels (97).

CPP 2 (CPPB2, OMIM #615346) is caused by heterozygous loss of function mutations in the *MKRN3/ZFP127* gene, located in the PWS critical region (chromosome 15q11-q13). An antisense RNA of unknown function overlaps this gene, probably regulating *MKRN3/ZFP127* expression. *MKRN3/ZFP127* is maternally imprinted and paternally expressed. Therefore only mutations inherited from fathers are disease-causing (97). It is noteworthy that a high frequency of *MKRN3/ZFP127* mutations was reported in a cohort of CCP males with anticipated puberty (98).

Puberty in humans normally starts when pulsatile GnRH is released from hypothalamic neurons. Indeed, the onset of puberty requires both a decrease in factors that inhibit the release of GnRH and an increase in stimulatory factors. MKRN3/ZFP127 protein levels declined prior to clinical onset of puberty and thereafter through puberty, which correlated negatively with gonadotropin concentrations in prepubertal girls (99) and its circulating levels declined during puberty in healthy boys (100). The expression pattern of *MKRN3/ZFP127* suggests the hypothesis of an inhibitory effect on GnRH secretion (101) but the precise mechanism by which its deficiency leads to an early reactivation of pulsatile GnRH secretion remains to be elucidated (95).

GnRH agonists have been the standard of care for the management of CPP in order to decrease bone maturation, growth velocity and progression of clinical signs of puberty (102).

## Chromosome 16

### Maternal Uniparental Disomy of Chromosome 16

UPD(16)mat has a high frequency since it is caused by trisomy 16 rescue (103). UPD(16)mat is associated with IUGR with an elevated risk of malformation but without a unique and specific phenotype. The heterogeneity of the phenotype suggests that placental insufficiency or mosaicism for trisomy 16 may be responsible for symptoms in such patients (36,104,105). Taken together, these data seem to indicate, as for UPD(6)mat, that a specific chromosome 16 associated imprinting disorder does not exist (105). On the other hand, some imprinted genes with unknown function have been identified on chromosome 16 and further studies are required to clarify the issue (106).

## Chromosome 20

### Pseudohypoparathyroidism

Pseudohypoparathyroidism (PHP) is a heterogeneous group of endocrine disorders characterized by renal resistance to parathyroid hormone (PTH), causing hypocalcaemia, hyperphosphatemia and elevated circulating PTH levels (3,107). Depending on the molecular defect, PHP includes other endocrine deficiencies related to hormone action resistance and other non-endocrine features. Overall, prevalence of PHP has been estimated to be 1.1 in 100,000 (108,109,110).

*GNAS* is a complex imprinting locus resulting in maternally, paternally, or biallelically expressed transcripts in differentially imprinted tissues: *Gs*α, the alpha-stimulatory subunit of the G protein; *XL*α*s*; *A/B*; *NESP*; and the antisense transcript *GNAS-AS1*. The antisense transcript* GNAS-AS1,* *A/B* and* XL*α*s *are transcribed from the paternal allele only; *NESP* is transcribed from the maternal allele only, and *Gs*α has a biallelical expression in most tissues, while its expression is restricted to the maternal allele in some others, including renal proximal tubule, thyroid, pituitary gland and gonads (111), even if the promoter of *Gs*α is not differentially methylated. The *GNAS* locus has two different IC regions (112); the first one is located within the *STX16* gene and controls the establishment of imprinting at the *GNAS A/B:TSS-DMR *only, while the second one, encompassing the antisense transcript* GNAS-AS1* on exons 3-4, controls the establishment of imprinting over the entire *GNAS* locus (111). Isolated imprinting defects at *GNAS A/B:TSS-DMR *are associated with deletions in the maternal allele affecting *STX16* and/or *NESP*, while overall imprinting alteration at the four DMRs of the *GNAS* locus is caused by maternal deletions at exons 3 and 4, or 40 and 33bp microdeletions at introns 4 and 3 of *GNAS-AS1* (3,111).

PHP type 1a (PHP1A, OMIM #103580) is caused by loss of function mutations in the maternal allele of *GNAS* gene. PHP1A patients present with generalized hormone resistance of variable degree, intellectual disability, obesity connected with decreased resting energy expenditure (113), and Albright hereditary osteodystrophy (AHO). AHO includes short stature, round facies, subcutaneous ossifications, brachydactyly and other skeletal anomalies (107).

Loss of function of *Gs*α on the paternal allele can cause pseudopseudohypoparathyroidism (PPHP) (OMIM #612463). Since renal tubular cells predominantly express the maternal allele of *GNAS*, a paternally inherited mutation results in a normal renal response to PTH, causing AHO without concurrent endocrine abnormalities (114). Paternal loss of function mutations can also cause progressive osseous heteroplasia (OMIM#166350), a condition characterized by subcutaneous ossifications presenting during childhood and progressing to involve subcutaneous and deep connective tissues, in the absence of AHO or hormone resistance (115).

Both PHP1A and PPHP individuals have halved *Gs*α expression in erythrocytes, which normally have a biallelic expression of *GNAS*. AHO may be caused by *Gs*α haploinsufficiency in tissues with *GNAS* biallelic expression (116).

In contrast, PHP type 1b (PHP1B, OMIM #603233) is clinically characterized by isolated renal PTH resistance and in some cases by thyroid stimulating hormone resistance. Rarely, these patients show an AHO phenotype (117). Interestingly, *Gs*α expression in erythrocytes is mildly reduced in patients with AHO (116). All patients with PHP1B have, at least, LoM at *GNAS* *A/B:TSS-DMR*, likely leading to the downregulated expression of the *GNAS-Gsa* transcript in imprinted tissues (111). Hormonal resistance is caused by LoM on the maternally inherited allele (118). Overall, 20% of PHP1B cases are inherited and caused by the previously mentioned deletions at the ICs, while the remaining 80% are sporadic and associated with methylation defects encompassing the whole *GNAS* locus. A small subset of the sporadic PHP1B cases is due to paternal UPD of chromosome 20q (6). Duplications and deletions in the *GNAS* locus have been identified in a few patients (119) but the majority of cases are still of unknown aetiology.

PHP patients should be screened for GH deficiency with the aim of eventually starting GH replacement therapy. Hypocalcaemia should be treated with an active form of vitamin D and calcium supplementation. Associated endocrinopathies, such as hypothyroidism and hypogonadism, should be treated. Surgical excision of AHO subcutaneous ossifications should only be considered in the presence of delimited, superficial lesions associated with pain and/or movement impairment (3).

## Maternal Uniparental Disomy of Chromosome 20

UPD(20)mat, generally caused by trisomy rescue after meiosis 2 nondisjunction, is characterized by IUGR, short stature and extreme feeding difficulties with failure to thrive from birth, often requiring gastric tube feeding in the first years of life. GH supplementation has been suggested as probably safe and effective for this condition (120). UPD(20)mat presents with phenotypic overlap with SRS, and must be considered in the SRS differential diagnosis (2).

## Conclusion

The imprinting disorders represent a rapidly evolving field in medicine and genetics. Their paradigm challenges traditional molecular diagnostic techniques and genetic counselling. A precise molecular diagnosis is essential and further clinical phenotyping is needed to provide the appropriate means for accurate management of these disorders.

Besides those described, it is likely that more imprinting disorders remain to be identified. This review briefly illustrated the rapidly evolving advances in the understanding of human genomic imprinting and related disorders. Novel discoveries in this field will likely occur in the next decade and will offer the potential for more precise molecular diagnosis and clinical definition, as well as the model for novel diagnostic and therapeutic techniques directed towards personalized medicine in the fields of growth, metabolism and cancer.

## Figures and Tables

**Table 1:**
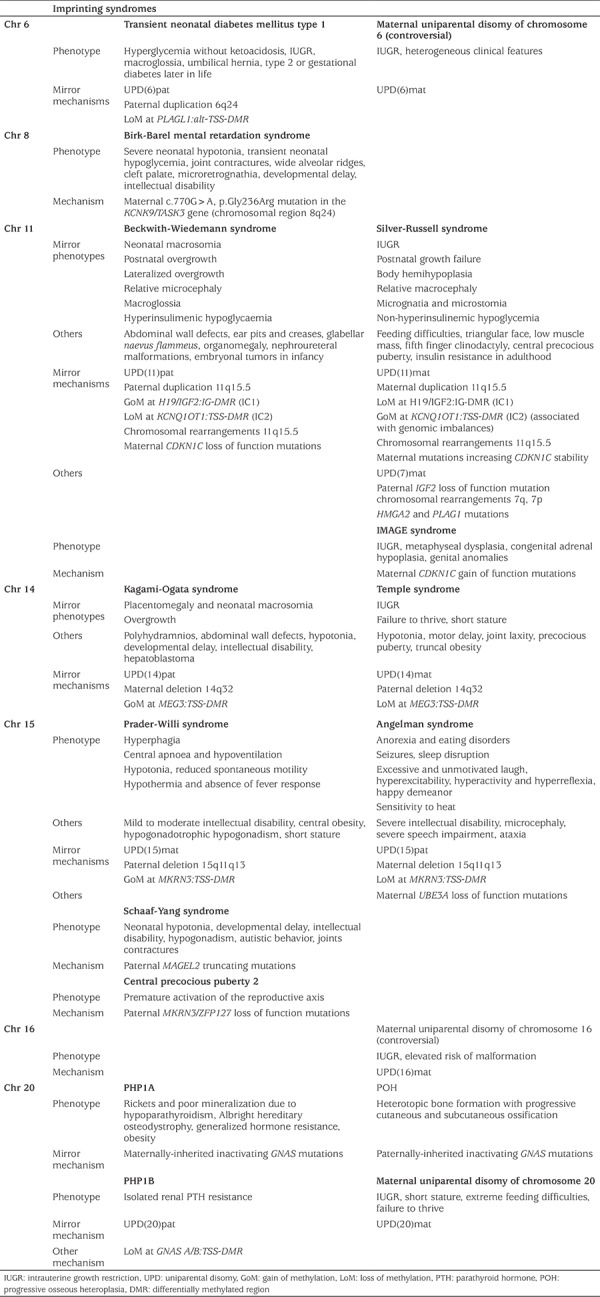
Summary of the clinical features and the molecular mechanisms of the human imprinting disorders
